# NMR Structural Study of the Prototropic Equilibrium in Solution of Schiff Bases as Model Compounds

**DOI:** 10.3390/molecules19010459

**Published:** 2013-12-31

**Authors:** David Ortegón-Reyna, Cesar Garcías-Morales, Itzia Padilla-Martínez, Efren García-Báez, Armando Aríza-Castolo, Ana Peraza-Campos, Francisco Martínez-Martínez

**Affiliations:** 1 Laboratorio de Posgrado, Facultad de Ciencias Químicas, Universidad de Colima, Km 9 Carretera Colima-Coquimatlán, Colima 28400, Mexico; E-Mails: dortegon@ucol.mx (D.O.-R.); peraza@ucol.mx (A.P.-C.); 2 Departamento de Química, Centro de Investigación y Estudios Avanzados del Instituto Politécnico Nacional, Av. IPN 2508, San Pedro Zacatenco 07360, México D. F., Mexico; E-Mails: cgarciam@cinvestav.mx (C.G.-M.); aariza@cinvestav.mx (A.A.-C.); 3 Departamento de Ciencias Básicas, Unidad Profesional Interdisciplinaria de Biotecnología del Instituto Politécnico Nacional, Av. Acueducto s/n Barrio la Laguna Ticomán, México D. F. 07340, Mexico; E-Mails: ipadillamar@ipn.mx (I.P.-M.); efren1003@yahoo.com.mx (E.G.-B.)

**Keywords:** Schiff bases, NHO prototropic tautomerism, NMR titration, δ-diagram

## Abstract

An NMR titration method has been used to simultaneously measure the acid dissociation constant (p*K*_a_) and the intramolecular NHO prototropic constant Δ*K_NHO_* on a set of Schiff bases. The model compounds were synthesized from benzylamine and substituted *ortho*-hydroxyaldehydes, appropriately substituted with electron-donating and electron-withdrawing groups to modulate the acidity of the intramolecular NHO hydrogen bond. The structure in solution was established by ^1^H-, ^13^C- and ^15^N-NMR spectroscopy. The physicochemical parameters of the intramolecular NHO hydrogen bond (*pK_a_*, Δ*K_NHO_* and ΔΔ*G*°) were obtained from ^1^H-NMR titration data and pH measurements. The Henderson–Hasselbalch data analysis indicated that the systems are weakly acidic, and the predominant NHO equilibrium was established using Polster–Lachmann δ-diagram analysis and Perrin model data linearization.

## 1. Introduction

Schiff bases are a great topic of basic research, that to date have an important place in organic chemistry and they have a great versatility in different fields of study. They have different biologic applications as antitumor agents [1,2,3,4,5], in the strengthening of immune response for cancer, in leukemia, in HIV, as anticonvulsant, antibacterials, antifungal, antiinflammatory, as prodrugs [6,7,8,9,10,11,12,13,14,15] and as study models in the intramolecular hydrogen bond from cofactor pyridoxal-5-phosphate [16,17,18,19,20]. They are also of interest because of their solvatochromic, thermochromic and photochromic properties with applications in optical recording technology, molecular electronics and photonics [21,22,23,24,25,26,27,28,29,30].

**Scheme 1:**
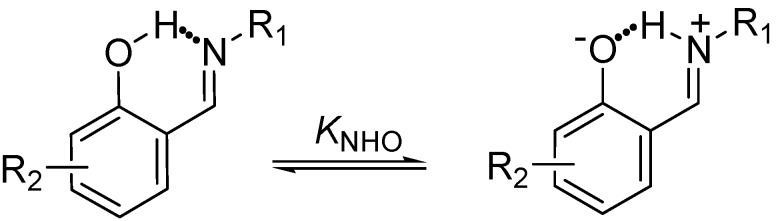
Prototropic 1,5 rearrangement in Schiff bases.

The Schiff bases derived from *ortho*-hydroxyaromatic aldehydes that are pentaconjugated non-symmetric systems [31] in which proton transfer from the oxygen hydroxyl to the nitrogen of imine, through the NHO hydrogen bond is observed (Scheme 1) have been extensively studied in recent years [32,33,34,35,36,37,38,39,40,41,42,43,44,45,46].

In these investigations several analytical methods for determining the prototropic equilibrium have been applied, such as FT-IR spectroscopy and X-ray diffraction in the solid state [45,46,47,48], as well as solution ^1^H-, ^13^C- and ^15^N-NMR [33,34,49,50,51]. This 1,5 tautomeric equilibrium is directly affected by the substituents [52,53,54,55,56] attached to both the phenyl group and the imine nitrogen which exert a strong influence on the acidity of the OH group, the basicity of the nitrogen atom and thus the NHO bond strength. Substituents also greatly increase the stability of the compounds by the effect of hydrogen bonding assisted by resonance (RAHB) [57,58,59]; preferences have been found in the position of the hydrogen either linked to oxygen (N∙∙∙H-O) or nitrogen (N-H∙∙∙O) [32,33,46,60] atoms and even being in the middle of both (O^−^∙∙∙H∙∙∙N^+^) [60,61].

For a prototropic acid-base-system HA in equilibrium, its equilibrium constant *K_a_* is expressed by Equation (1), which after logarithms becomes the Henderson-Hasselbalch Equation (2):

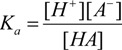
(1)

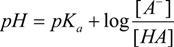
(2)


Equation (2) is directly related to the chemical shifts of active nuclei in NMR, which are dependent on *pH* changes, this leads to Equation (3):

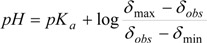
(3)


The *pK_a_* is experimentally obtained, using the tabulation of log[(δ_max_ − δ_obs_)/(δ_obs_ − δ_min_)] against pH, where δ_min_ and δ_max_ are the chemical shifts in the inflection points in the titration curve, while δ_obs_ is the observed chemical shift during the course of the titration, so the equilibrium point is at point zero, which corresponds to *pH* = *pKa* [44,62,63,64,65,66,67]. This method has been extensively used because of its simplicity, however is limited by variability in *pH* readings and accuracy in measuring the volumes of the titrant.

Polster and Lachmann postulated the Gibbs triangle method, which later emerged as the absorbance diagram (A-diagram) or chemical shift diagram (δ-diagram), depending on the spectrometry used for the analysis of data from a titration, for the study of acid-base systems [62,68]. This method allows the evaluation of the quotient of acidity constants (*ΔK_a_*) of two or more compounds, mainly in diprotic and polyprotic acid-base systems [68], on the bases of a ratio of distances from the Gibbs triangle which is independent of *pH* readings [68].

Later, Perrin *et al.* [69,70,71,72] also developed a mathematical model for the determination of *ΔK_a_* for mixtures of isomers in equilibrium with independency from the *pH* readings by drawing δ-diagrams also, so this model can be applied to the analysis of acid-base equilibrium mainly in monoprotic systems. Then for two acids HA and HB, the quotient of their acidity constants *ΔK_a_*, can be measured by the variation in chemical shifts due to changes in the acidity of the systems:

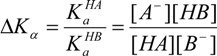
(4)


Equation (4), written in terms of chemical shifts when *ΔK_a_* ≠ 1, allows the evaluation of *ΔK_a_* as the slope of a straight line, Equation (5):

(*δ_b_ − δ_B°_*)(*δ_AH_ − δ_a_*) = ∆*K_α_*(*δ_a_ − δ_A°_*)(*δ_BH_ − δ_b_*) (5)

where δ_A°_, δ_B°_ are the chemical shifts from species at the start of the titration, δ_a_, δ_b_ are the chemical shifts observed during the titration, andδ_HA_, δ_HB_ are the chemical shifts from species at the end of the titration.

**Figure 1:**
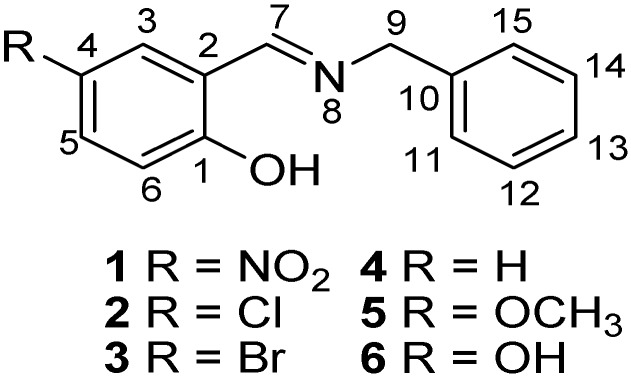
Schiff base derivatives of **1**–**6**.

In this contribution, both the Perrin and Polster-Lachmann models are applied to the study of intramolecular hydrogen bonds that involve prototropic equilibrium with the aim to find with accuracy and selectivity the position of the proton on the oxygen or nitrogen atoms. The model compounds were a set of Schiff base derivatives of 5-nitrosalicylaldehyde, 5-chlorosalicylaldehyde, 5-bromo-salicylaldehyde, salicylaldehyde, 5-methoxysalicyladehyde and 5-hydroxysalicyladehyde with benzylamine (compounds **1**–**6**, Figure 1). The substituents were selected in order to cover a broad range of both electrodonating (ED) and electrowithdrawing (EW) groups whose electronic effects could modulate the NHO hydrogen bonding scheme. ^1^H-NMR spectrometry was used as the titration method.

## 2. Results and Discussion

### 2.1. NMR Spectra

Synthesized compounds were identified by ^1^H-, ^13^C- and ^15^N-NMR. The ^1^H-NMR spectra of compounds **1**–**6** in DMSO-*d_6_* solution showed remarkable changes in the chemical shift of the acidic proton NHO in the range of 12.53–14.34 ppm, in response to the electronic character of the substituent R. Since a larger value in the chemical shift indicates a greater acidity of the proton, compound **1** has the largest acidity and compound **6** has the lowest acidity. Simultaneously the chemical shift of protons H3, H5, H6 and H7 were affected too.

**Scheme 2:**
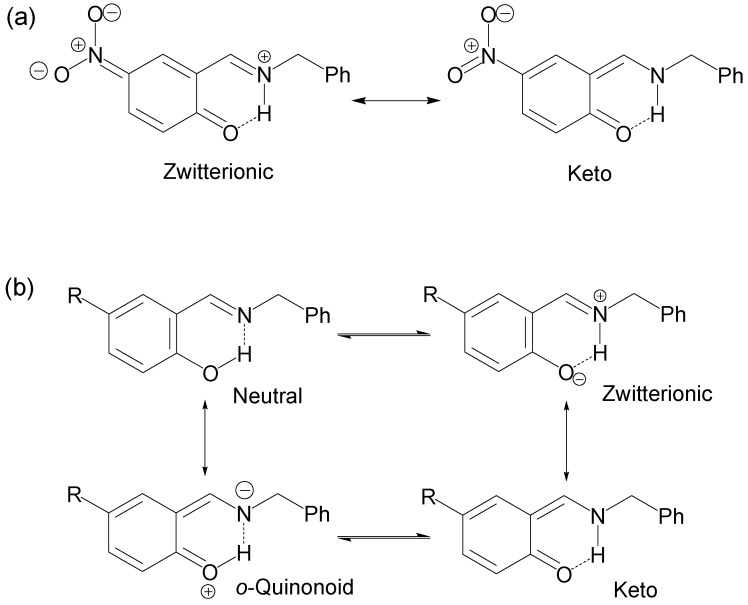
Possible resonance and equilibrium structures for compounds **1**–**6**.

The ^13^C spectra of all compounds showed clear shielding and deshielding effects, according to the substituent, mainly from C1 to C7. The chemical shifts of compound **1** were more affected than those of compounds **2**–**6**, especially the carbon atoms C1 and C4. Compound **1**, the NO_2_ derivative, showed a chemical shift of 175.8 and 136.9 ppm for C1 and C4, respectively, where C1 is in the range of carbonyl chemical shifts (170 to 200 ppm) while C4 is in the range of nitro Schiff base compounds (130 to 150 ppm). The ^15^N chemical shift of compound **1** was −162.1 ppm, indicating an average between imine-enamine forms, therefore in this last compound the zwitterionic structure (Scheme 2a) is favored and the hydrogen H8 is localized with the nitrogen atom (^+^N–H∙∙∙O).

In the case of compounds **2**–**6** the chemical shifts of C1 appear at lower frequencies from 160.4 to 153.5 ppm, a region characteristic of OH structures (150–160 ppm) and the chemical shifts of the imine C7=N appear from 165.8 to 167.4 ppm, a less significant variation. The ^15^N chemical shifts for compounds **2**–**6** were in the range of −79.7 to −81.8 ppm (−50 to −90 ppm for imine), in agreement with a neutral N∙∙∙H–O tautomeric form with the hydrogen H8 is localized with the oxygen atom (Scheme 2b). The NMR chemical shifts of compounds **1**, **3**, **4** and **5** have already been reported [73] and are in agreement with the above mentioned results, except for the nitro derivative **1** for which the authors conclude that the N–H tautomer is present in solution instead of the zwitterion form proposed herein.

### 2.2. NMR Titration

All compounds were titrated in CD_3_OD solution with NaOD, and only compound **2** was further titrated with DCl. ^1^H-NMR spectra were recorded after each aliquot of titrant and simultaneously the *pH* was measured following each recorded spectrum. The resonances of H6 and H9 were used to plot *pH vs.* δ^1^H, because these protons were most affected by deprotonation of the labile hydrogen.

**Figure 2:**
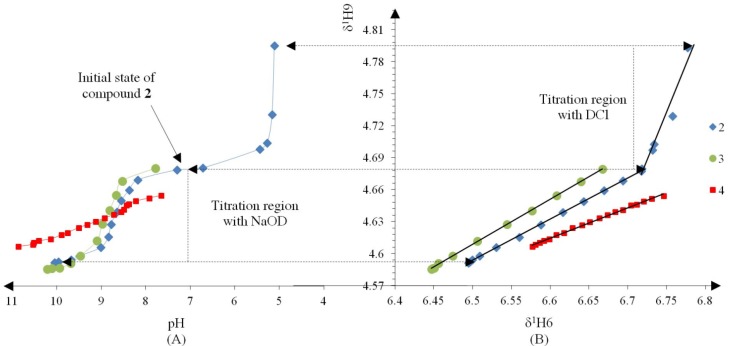
(**A**) Titration curve of compounds **2**–**4**; compound **2** was titrated with DCl; only the titration region with NaOD was used to calculate the *pK_a_* values with the Henderson-Hasselbalch equation; (**B**) δ-Diagram of δ^1^_H6_ *vs.* δ^1^_H9_ of compounds **2**–**4**; the initial data obtained was not linearized but after using the Perrin model the data became for linearized compounds **2** (Cl), **3** (Br) and **4** (H) to obtain the slope *ΔK_NHO_* as shown in Scheme 3.

Compound **2** was initially titrated with DCl, however hydrolysis occured with the acid titrant and only five ^1^H spectra and their corresponding *pH* readings could be recorded, so subsequently all compounds were titrated with NaOD (Figure 2A).

**Figure 3:**
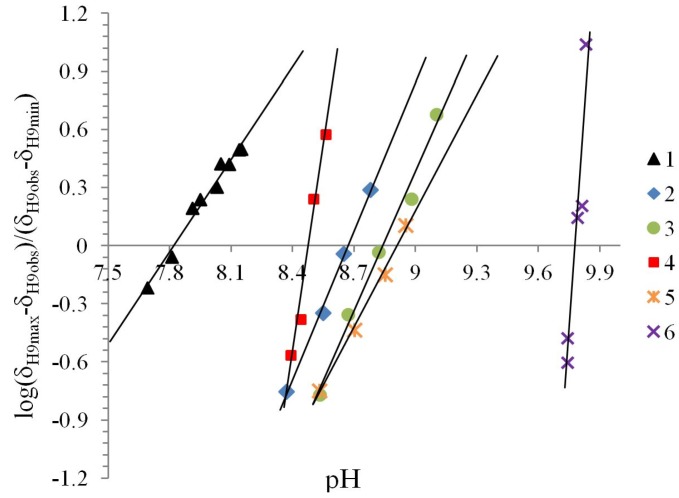
The experimental *pK_a_* were found using the plot of the Henderson-Hasselbalch equation, when *log*[(δ_H9max_ − δ_H9obs_)/(δ_H9obs_ − δ_H9min_)] = 0 then the *pH* intercept is the *pK_a_* value.

The Henderson-Hasselbalch equation was used to measure *pK_a_* values of the compounds by a graphical method with plots of *pH* against *log*[(δ_H9max_ − δ_H9obs_)/(δ_H9obs_ − δ_H9min_)] (Figure 3) from the titration curve, while *ΔK_NHO_* was obtained from the δ-diagram (Figure 2B) using the Perrin model linearization [(δ_H9_ − δ_H9°_)(δ_H6e_− δ_H6_) *vs.* (δ_H6_ − δ_H6°_)(δ_H9e_− δ_H9_)] for compounds **2**, **3**, **4** and by Polster and Lachmann analysis for compounds **1**, **5** and **6**. Table 1 summarizes the physicochemical parameters obtained by the graphical methods mentioned above.

**Table 1:** Physicochemical parameters of compounds **1**–**6** at 296.15 ± 1 K in CD_3_OD solution.

Compound	*K_a_*/10^−9^	*pK_a_*	*ΔK_NHO_*	*ΔpK_NHO_*	*ΔΔG°* ^[a]^
1	15.1	7.8	1.04(±0.05)	−0.017	−0.097
2	2.13	8.6	1.031(±0.002)	−0.0133	−0.075
3	1.44	8.8	0.986(±0.002)	0.006	0.036
4	3.33	8.4	0.841(±0.005)	0.0754	0.426
5	1.23	8.9	1.021(±0.014)	−0.01	−0.052
6	0.17	9.7	1.02(±0.02)	−0.001	−0.004

^[a]^ ***ΔΔ****G°* = −*RTln****Δ****K_NHO_* (kJ mol^−1^ K^−1^).

The *pK_a_* values obtained by the Henderson-Hasselbalch equation for all compounds were greater than 7 and less than 11, showing that these compounds are weak acids, the *pK_a_* value increases in the order NO_2_ < H < Cl < Br < OMe < OH. Only compound **6** showed two *pK_a_* values, the value of 9.7 belongs to NHO and the second value of 9.8 to the phenolic hydroxyl group C4-OH. On the other hand the *ΔΔG°* values, associated with the prototropic NHO equilibrium, are favored in the order NO_2_ > Cl > OMe ≈ OH > Br > H.

Figure 2 shows the titration curve with full pH scale (A) and the δ-diagram (B) of compounds **2** to **4**; only the data region titrated with NaOD was taken for the *pK_a_* value calculation. All compounds should show the same shape of δ-diagram as they were titrated with NaOD. However, the titration curves of compounds **2**, **3** and **4** showed an almost linear behavior, whereas those of compounds **1** and **5** showed one inflection point and those of compound **6** two inflection points (see Supporting Information). These results indicate that the initial structure of compounds **1**–**6**, at the beginning of the titration, was not the same in agreement with the NMR data discussed above.

**Scheme 3:**
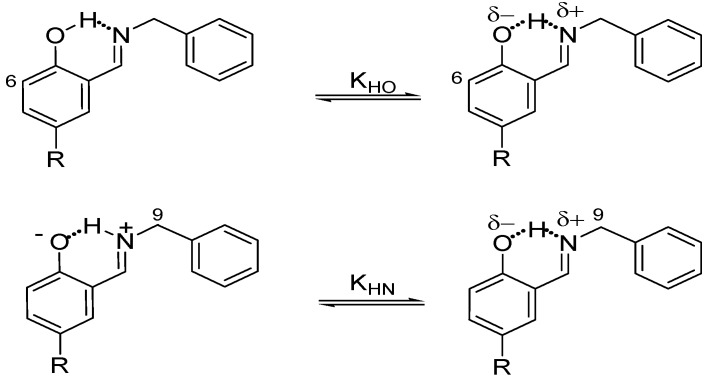
Equilibria variation in the prototropic 1,5-tautomeric equilibrium. The quotient of the equilibrium constants *K*_HN_ and *K*_HO_ is *ΔK_NHO_*.

On the other hand, the prototropic 1,5-rearrangement (Scheme 3) can be envisaged as composed by two equilibria as depicted by in Figure 2. The quotient of the equilibrium constants *K*_HN_ and *K*_HO_ is defined as *ΔK_NHO_*, corresponding to the equilibrium constant of the prototropic 1,5-tautomerism. The chemical shifts of H6 and H9 are the most sensitive to changes in the equilibrium positions, thus they were used as probes for *K*_HO_ and *K*_HN_ measurements, respectively.

Thus, the *ΔK_NHO_* value allows one to establish the position of the NHO equilibrium. Therefore, if the *ΔK_NHO_* value is equal to 1 then the system is in equilibrium N^δ+^∙∙∙H∙∙∙O^δ−^ and both *ΔpK_NHO_* and *ΔΔG°* are equal to zero; if the value of *ΔK_NHO_* is higher than 1, both *ΔpK_NHO_* and *ΔΔG°* are less than zero and labile hydrogen is predominantly positioned on the N atom, ^+^N–H∙∙∙O; and finally if *ΔK_NHO_* is less than 1 then *ΔpK_NHO_* and *ΔΔG°* are greater than zero and therefore the labile hydrogen is predominantly positioned on the O atom, N∙∙∙H–O.

**Figure 4:**
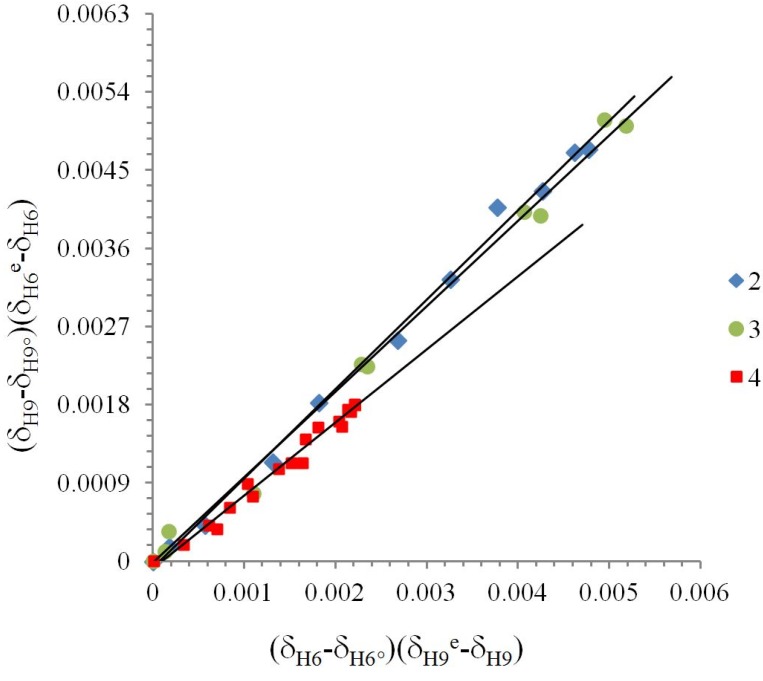
Linearization of ^1^H chemical shifts from the δ-diagram for compounds **2**, **3** and **4** by Perrin model. (δ_H9_ − δ_H9°_)(δ_H6e_ − δ_H6_) = *ΔK_NHO_* (δ_H_ − δ_H6°_)(δ_H9e_ − δ_H9_), *ΔK_NHO_* is the slope of the straight line. Compounds **2** **(Cl)** *y* = 1.031(±0.002)*x* + 8*10^−5^, R = 0.996; **3** (Br) *y* = 0.986(±0.002)*x* − 2*10^−5^, R = 0.994; **4** (H) *y* = 0.841(±0.005)*x* − 9*10^−5^, R = 0.983.

**Figure 5:**
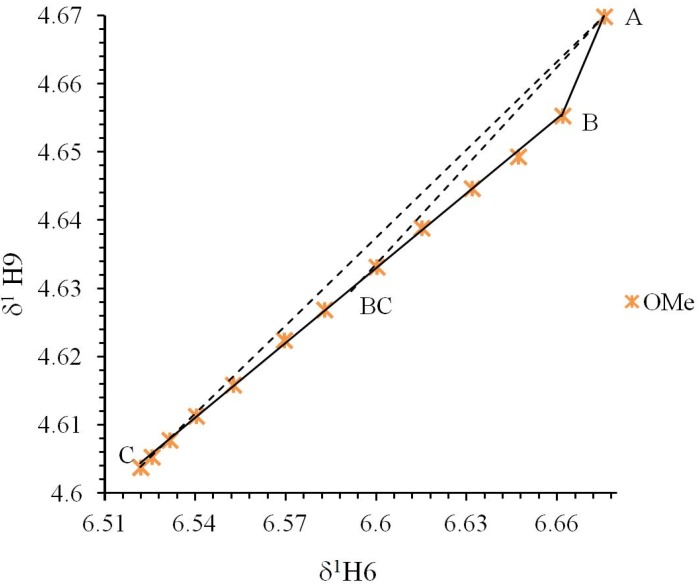
Polster-Lachmann analysis of the δ-diagram from compound **5**. Point “A” shows the initial state, point “B” indicates a change on the compound (neutralization point), point “C” the final state and point “BC” is the experimental *ΔK_NHO_* of the system. The dotted line A to C shows the shape of a triangle; the dotted line from vertex A to point BC shows the intercept in an *ΔK_NHO_* equilibrium point.

Compounds **2**, **3** and **4** were treated with the Perrin model (Figure 4) for the nonlinear behavior in the δ-diagram. In the case of compounds **1**, **5** and **6** the Polster-Lachmann analysis seems to be more appropriate, because of the shape of the Gibbs triangle taken in the δ-diagram (Figure 5).

The Polster-Lachmann analysis is based on a ratio of distances established by the Gibbs triangle and the *ΔK_NHO_* values are obtained from the chemical shifts of the titration data, hence from points A, B, C and BC in the δ-diagram, in agreement with Equation (6):

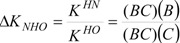
(6)


**Scheme 4:**
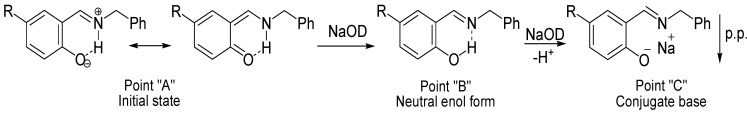
Mechanism proposed during the titration with NaOD; the scheme is according to points in the δ-diagram of Figure 5.

From δ-diagrams, the mechanism occurring in the course of the titration with NaOD, can be proposed (Scheme 4).

Compounds **1**, **2**, **5** and **6** begin at an initial state as acidic species (Point “A”), with a small increase of *pH*, the intramolecular hydrogen bond equilibrium is shifted from ^+^N-H∙∙∙O^−^ to N∙∙∙H-O, reaching the neutralization point of the solution (Point “B”); then, as long as the *pH* is increased compounds are deprotonated to become into the conjugated bases that precipitate as a salt (Point “C”). In the case of compounds **3** and **4**, the initial state is at point “B” with the intramolecular hydrogen bond in the N∙∙∙H-O form, the addition of NaOD aliquots only shift the equilibrium to point “C” the conjugate base.

The δ-diagrams show the initial state in all compounds and indicate the most stable species in a methanol solution, so the stability of the NHO intramolecular hydrogen bond is affected by the electronic nature of the substituent as well as solvation of methanol; therefore, the structure of compounds **1**, **5** and **6** with NO_2_, OMe and OH substituents, respectively, stabilizes and direct the NHO equilibrium position by both mesomeric and inductive effects, although they have different *ΔK_NHO_* values, whereas the halogen substituent in compounds **2** and **3** exert both electronegative and inductive effects; none of such effects are present in compound **4**. Thus from the obtained *ΔK_NHO_* values, the predominant NHO equilibrium in compounds **3** and **4** are the neutral N∙∙∙H–O form, while for the rest of the compounds the zwitterionic ^+^N–H∙∙∙O^−^ form is present (Scheme 2). In the particular case of compound **1**, it is as an imine-enamine tautomeric form in agreement with ^1^H, ^13^C and ^15^N pfg-HMQC spectroscopy mentioned above.

Finally, the obtained *ΔK_NHO_* values (Table 1) are very close to the equilibrium point N^δ−^∙∙∙H^+^∙∙∙O^δ−^ (*ΔK_NHO_* = 1), which indicate a fast interchange of intramolecular hydrogen bond and the effect produced by both the substituent and the solvent that stabilize the systems in a preferred tautomeric form.

## 3. Experimental

### 3.1. General Remarks

**Scheme 5:**
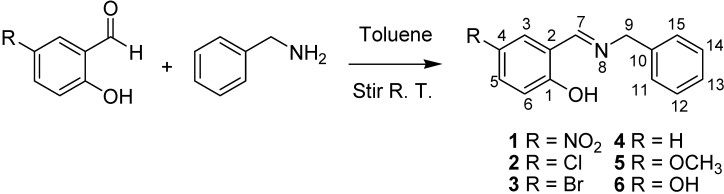
Synthesis of Schiff bases.

Schiff bases **1** to **6** were obtained by condensation of the appropriate aromatic *ortho-*hydroxyaldehyde with benzylamine in toluene at 25 °C (Scheme 5). Solids products were filtered and dried under a *vacuum*. Compound **4** was a liquid and the excess of toluene was eliminated under vacuum. ^1^H and ^13^C-NMR spectra were recorded in DMSO-*d*_6_ on a JEOL ECA-500 spectrometer (^1^H, 500.1 MHz; ^13^C, 125.7 MHz; ^15^N, 50.7 MHz) and the ^15^N chemical shifts were obtained by correlation of ^1^H, ^15^N pfg-HMQC (see Supporting Information).

*(E)-2-((Benzylimino)methyl)-4-nitrophenol* (**1**). Compound **1** was prepared as reported [73] by condensation of 5-nitrosalicylaldehyde (0.5 g, 2.99 mmol) with benzylamine (0.32 g, 0.32 mL, 2.99 mmol) in toluene at room temperature (25 °C) and with a stirring time of 5 min. ^1^H-NMR (DMSO-*d*_6_): δ = 8.42 (*d*, ^4^*J*_H,H_ = 2.9 Hz, 1H, H3), 8.02 (*dd*, ^3^*J*_H,H_ = 9.5, ^4^*J*_H,H_ = 3.0 Hz, 1H, H5), 6.65 (*d*, ^3^*J*_H,H_ = 9.5 Hz, 1H, H6), 8.86 (*s*, 1H, H7), 4.84 (*s*, 2H, H9), 7.28–7.37 (*m*, 5H, H11-H15), 14.3 (broad signal, 1H, NHO) ppm. ^13^C-NMR (DMSO-*d*_6_): δ = 175.8 (*s*, 1C, C1), 115.0 (*s*, 1C, C2), 132.1 (*s*, 1C, C3), 136.9 (*s*, 1C, C4), 129.5 (*s*, 1C, C5), 122.2 (*s*, 1C, C6), 167.4 (*s*, 1C, C7), 57.2 (*s*, 1C, C9), 135.5 (*s*, 1C, C10), 129.3 (*s*, 2C, C11, C15), 128.6 (*s*, 2C, C12, C14), 128.5 (*s*, 1C, C13) ppm. ^15^N-NMR (DMSO-*d*_6_): δ = −162.1 (*s*, 1N, N8), −9.5 (*s*, 1N, N4) ppm.

*(E)-2-((Benzylimino)methyl)-4-chlorophenol* (**2**)*.* Compound **2** was prepared by condensation of 5-chlorosalicylaldehyde (0.5 g, 3.19 mmol) with benzylamine (0.34 g, 0.34 mL, 3.19 mmol) in toluene at room temperature (25 °C) and with a stirring time of 5 min. Yield 0.73 g (93%). m.p. 359–360 K. FT-IR (ATR, cm^−1^): 1628 (C=N), 1573 (asymmetrical C=C-O-H stretch), 1479 (symmetrical C=C-O-H stretch), 3069 (intramolecular hydrogen bonding N∙∙∙H-O, as a weak broad band). LC-MS-TOF in HPLC-methanol solution, *m*/*z* (%) calculated: 246.0686 (100); found: 246.0680 (100) [M+H]^+^, empirical formula C_14_H_13_NOCl. ^1^H-NMR (DMSO-*d*_6_): δ = 7.53 (*d*, ^4^*J*_H,H_ = 2.7 Hz, 1H, H3), 7.31 (*dd*, ^3^*J*_H,H_=8.7, ^4^*J*_H,H_ = 2.7 Hz, 1H, H5), 6.87 (*d*, ^3^*J*_H,H_ = 8.8 Hz, 1H, H6), 8.64 (*s*, 1H, H7), 4.76 (*s*, 2H, H9), 7.23–7.34 (*m*, 5H, H11-H15), 13.4 (broad signal, 1H, NHO) ppm. ^13^C-NMR (DMSO-*d*_6_): δ = 160.0 (*s*, 1C, C1), 120.2 (*s*, 1C, C2), 131.1 (*s*, 1C, C3), 122.5 (*s*, 1C, C4), 132.5 (*s*, 1C, C5), 119.0 (*s*, 1C, C6), 165.8 (*s*, 1C, C7), 62.4 (*s*, 1C, C9), 138.8 (*s*, 1C, C10), 129.1 (*s*, 2C, C11, C15), 128.3 (*s*, 2C, C12, C14), 127.7 (*s*, 1C, C13) ppm. ^15^N-NMR (DMSO-*d*_6_): δ = −80.5 (*s*, 1N, N8) ppm.

*(E)-2-((Benzylimino)methyl)-4-bromophenol* (**3**). Compound **3** was prepared as reported [73] by condensation of 5-bromosalicylaldehyde (0.5 g, 2.48 mmol) with benzylamine (0.26 g, 0.27 mL, 2.48 mmol) in toluene at room temperature of 25 °C and with a stirring time of 5 min. ^1^H-NMR (DMSO-*d*_6_): δ = 7.64 (*d*, ^4^*J*_H,H_ = 2.5 Hz, 1H, H3), 7.41 (*dd*, ^3^*J*_H,H_ = 8.7 and ^4^*J*_H,H_ = 2.7 Hz, 1H, H5), 6.82 (*d*, ^3^*J*_H,H_ = 8.7 Hz, 1H, H6), 8.62 (*s*, 1H, H7), 4.75 (*s*, 2H, H9), 7.22–7.33 (*m*, 5H, H11-H15), 13.5 (broad signal, 1H, NHO) ppm. ^13^C-NMR (DMSO-*d*_6_): δ = 160.4 (*s*, 1C, C1), 120.9 (*s*, 1C, C2), 134.0 (*s*, 1C, C3), 109.8 (*s*, 1C, C4), 135.3 (*s*, 1C, C5), 119.5 (*s*, 1C, C6), 165.7 (*s*, 1C, C7), 62.4 (*s*, 1C, C9), 138.8 (*s*, 1C, C10), 129.1 (*s*, 2C, C11, C15), 128.3 (*s*, 2C, C12, C14), 127.8 (*s*, 1C, C13) ppm. ^15^N-NMR (DMSO-*d*_6_): δ = −81.8 (*s*, 1N, N8) ppm.

*(E)-2-((Benzylimino)methyl)phenol* (**4**). Compound **4** was prepared as reported [73] by condensation of salicylaldehyde (0.5 g, 0.42 mL, 4.09 mmol) with benzylamine (0.43 g, 0.44 mL, 4.09 mmol) in toluene at room temperature (25 °C) and with a stirring time of 5 min. ^1^H-NMR (DMSO-*d*_6_): δ = 7.44 (*dd*, ^3^*J*_H,H_ = 7.5, ^4^*J*_H,H_ = 1.7 Hz, 1H, H3), 6.87 (*td*, ^3^*J*_H,H_ = 7.4, ^3^*J*_H,H_ = 8.3 and ^4^*J*_H,H_ = 0.9, 1H, H4), 7.30 (*t*, *d*, ^3^*J*_H,H_ = 7.4, ^3^*J*_H,H_ = 8.2 and ^4^*J*_H,H_ = 1.7 Hz, 1H, H5), 6.85 (*dd*, ^3^*J*_H,H_ = 8.2 and ^4^*J*_H,H_ = 1.0 Hz, 1H, H6), 8.67 (*s*, 1H, H7), 4.76 (*s*, 2H, H9), 7.23–7.34 (*m*, 5H, H11-H15), 13.4 (*s*, 1H, NHO) ppm. ^13^C-NMR (DMSO-*d*_6_): δ = 161.2 (*s*, 1C, C1), 119.2 (*s*, 1C, C2), 132.3 (*s*, 1C, C3), 119.1 (*s*, 1C, C4), 132.9 (*s*, 1C, C5), 117.0 (*s*, 1C, C6), 166.8 (*s*, 1C, C7), 62.7 (*s*, 1C, C9), 139.0 (*s*, 1C, C10), 129.0 (*s*, 2C, C11, C15), 128.2 (*s*, 2C, C12, C14), 127.6 (*s*, 1C, C13) ppm. ^15^N-NMR (DMSO-*d*_6_): δ = −81.7 (*s*, 1N, N8) ppm.

*S1-5 (E)-2-((Benzylimino)methyl)-4-methoxyphenol* (**5**). Compound **5** was prepared as reported [73] by condensation of 5-methoxysalicylaldehyde (0.5 g, 0.40 mL, 3.28 mmol) with benzylamine (0.35 g, 0.35 mL, 3.28 mmol) in toluene at room temperature (25 °C) and with a stirring time of 5 min. ^1^H-NMR (DMSO-*d*_6_): δ = 7.04 (*d*, ^4^*J*_H,H_ = 3.1 Hz, 1H, H3), 6.95 (*dd*, ^3^*J*_H,H_ = 9.0 and ^4^*J*_H,H_ = 3.1 Hz, 1H, H5), 6.87 (*d*, ^3^*J*_H,H_ = 9.0, 1H, H6), 8.58 (*s*, 1H, H7), 4.74 (*s*, 2H, H9), 7.22–7.34 (*m*, 5H, H11-H15), 3.70 (*s*, 3H, H16), 12.8 (*s*, 1H, NHO) ppm. ^13^C-NMR (DMSO-*d*_6_): δ= 155.0 (*s*, 1C, C1), 119.0 (*s*, 1C, C2), 115.4 (*s*, 1C, C3), 152.2 (*s*, 1C, C4), 119.8 (*s*, 1C, C5), 117.7 (*s*, 1C, C6), 166.6 (*s*, 1C, C7), 62.9 (*s*, 1C, C9), 139.1 (*s*, 1C, C10), 129.0 (*s*, 2C, C11, C15), 128.2 (*s*, 2C, C12, C14), 127.6 (*s*, 1C, C13), 55.9 (*s*, 1C, C16) ppm. ^15^N-NMR (DMSO-*d*_6_): δ = −79.8 (*s*, 1N, N8) ppm.

*(E)-2-((Benzylimino)methyl)benzene-1,4-diol* (**6**). Compound **6** was prepared by condensation of 5-hydroxysalicylaldehyde (0.5 g, 3.62 mmol) with benzylamine (0.38 g, 0.39 mL, 3.62 mmol) in toluene at room temperature of 25 °C and with a stirring time of 5 min. Yield 0.73 g (89%). m.p. 397–399 K. FT-IR (ATR, cm^−1^): 1641 (C=N), 1601 (asymmetrical C=C-O-H stretch), 1496 (symmetrical C=C-O-H stretch), 3311 (free phenolic O-H medium broad band), 3054 (intramolecular hydrogen bonding N∙∙∙H-O, as a weak broad band). LC-MS-TOF in HPLC-grade methanol, *m*/*z* (%) calculated: 228.1025 (100); found: 228.1022 (100) [M+H]^+^, empirical formula C_14_H_14_NO_2_. ^1^H-NMR (DMSO-*d*_6_): δ= 6.83 (*d*, ^4^*J*_H,H_ = 3.0 Hz, 1H, H3), 6.76 (*dd*, ^3^*J*_H,H_ = 8.8, ^4^*J*_H,H_ = 3.0 Hz, 1H, H5), 6.68 (*d*, ^3^*J*_H,H_ = 8.9, 1H, H6), 8.58 (*s*, 1H, H7), 4.74 (*s*, 1H, H9), 7.23–7.34 (*m*, 5H, H11-H15), 9.00 (*s*, 1H, C4-OH), 12.5 (*s*, 1H, NHO) ppm. ^13^C-NMR (DMSO-*d*_6_): δ= 153.5 (*s*, 1C, C1), 119.1 (*s*, 1C, C2), 117.0 (*s*, 1C, C3), 149.9 (*s*, 1C, C4), 120.5 (*s*, 1C, C5), 117.4 (*s*, 1C, C6), 166.8 (*s*, 1C, C7), 62.8 (*s*, 1C, C9), 139.3 (*s*, 1C, C10), 129.1 (*s*, 2C, C11, C15), 128.3 (*s*, 2C, C12, C14), 127.6 (*s*, 1C, C13) ppm. ^15^N-NMR (DMSO-*d*_6_): δ= −79.7 (*s*, 1N, N8) ppm.

### 3.2. Sample Preparation, Titrant Solution and pH Meter

Solutions of compounds **1**–**6** (0.06–0.10 M) in CD_3_OD (0.4–0.5 mL) and 1,4-dioxane as internal reference (0.5–1.5 μL, δ_H_ 3.53), were prepared in resonance tubes. The NaOD titrant solution, was prepared to 1.4 and 4.8% (v/v) from NaOD/D_2_O (40%) in CD_3_OD, while the DCl solution was prepared to 5% from DCl/D_2_O (70%) in CD_3_OD. The glass electrode was filled with a KCl standard solution and calibrated with phosphate buffer pH 7.0 and 4.0.

### 3.3. NMR Spectrometric Titration

The ^1^H-NMR spectra were recorded in CD_3_OD on a JEOL ECA-500 spectrometer at room temperature of 295.15 ± 1 K (22 ± 1 °C). An initial ^1^H-NMR spectrum of the solutions was recorded and assigned as initial value for the titration. Subsequently the solutions were titrated with aliquots of the NaOD/D_2_O solution base (3.0 μL), until invariant changes in the chemical shifts were observed; each ^1^H-NMR spectrum as well as the corresponding *pH* reading were recorded simultaneously, after the addition of the base. Only compound **2** was further titrated with DCl (5%), to observe the behavior of the system at acidic pH.

### 3.4. NMR Titration Graphics (Figure 6, Figure 7, Figure 8, Figure 9, Figure 10 and Figure 11)

**Figure 6:**
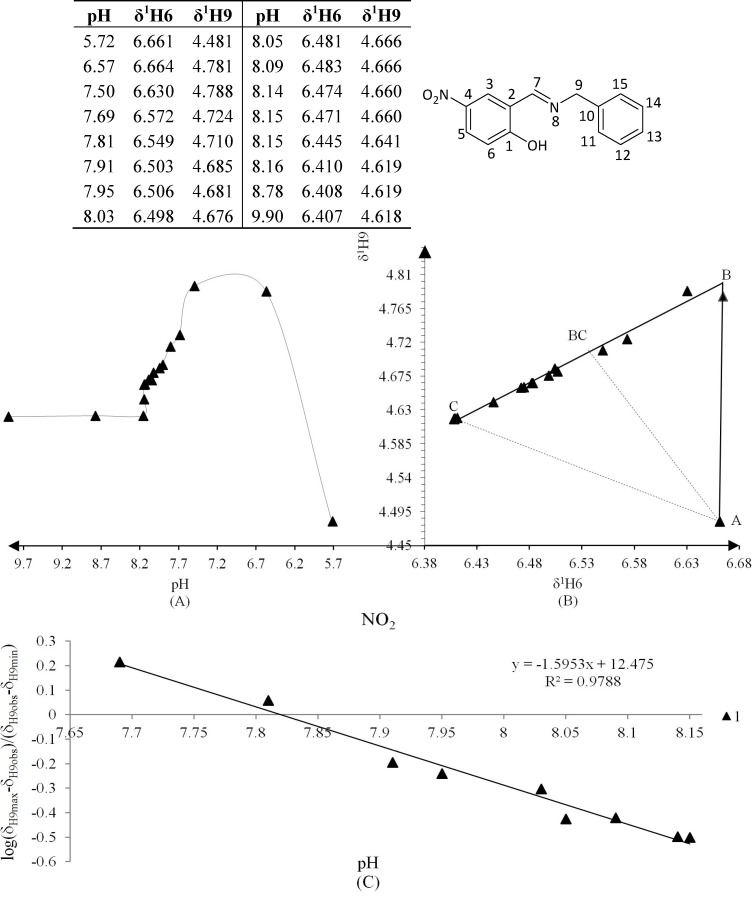
(**A**) titration curve (pH *vs.* δ^1^H9); (**B**) δ-diagram with Polster-Lachmann analysis (δ^1^H6 *vs.* δ^1^H9) and (**C**) plot of the semilogarithmic Henderson-Hasselbalch equation (pH *vs.* log[(δ_H9max_ − δ_H9obs_)/(δ_H9obs_ − δ_H9min_)]) of compound **1** (R = NO_2_).

**Figure 7:**
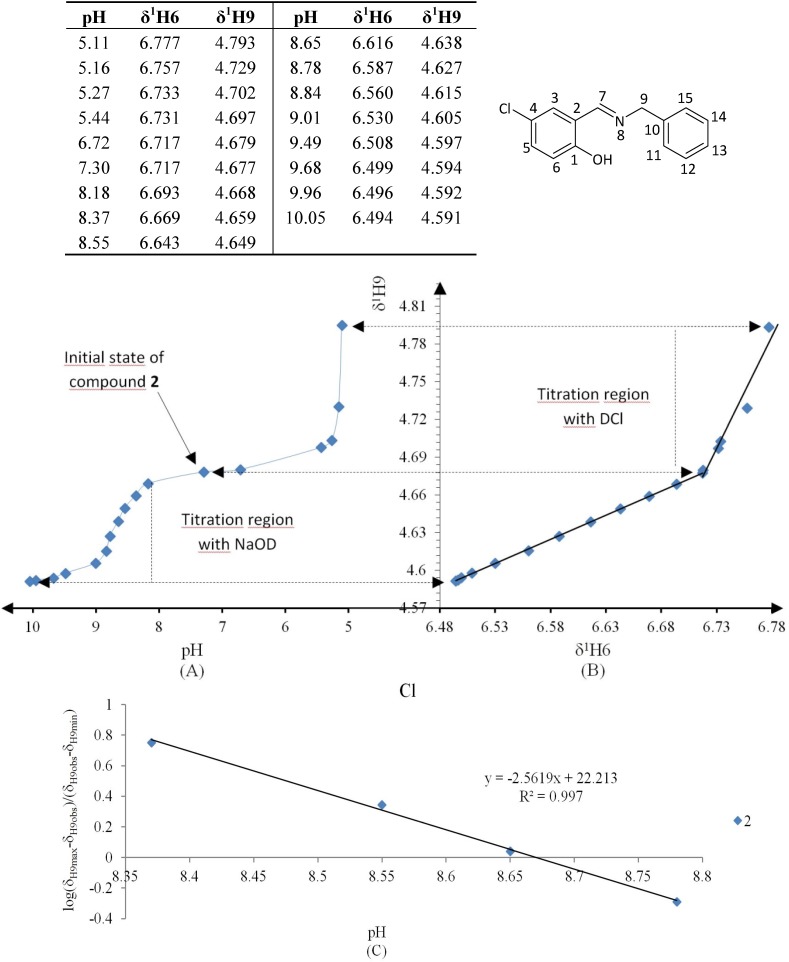
(**A**) titration curve (pH *vs.* δ^1^H9); (**B**) δ-diagram with Perrin model analysis (δ^1^H6 *vs.* δ^1^H9) and (**C**) plot of the semilogarithmic Henderson-Hasselbalch equation (pH *vs.* log[(δ_H9max_ − δ_H9obs_)/(δ_H9obs_ − δ_H9min_)]) of compound **2** (R = Cl). Only compound **2** was titrated with DCl solution.

**Figure 8:**
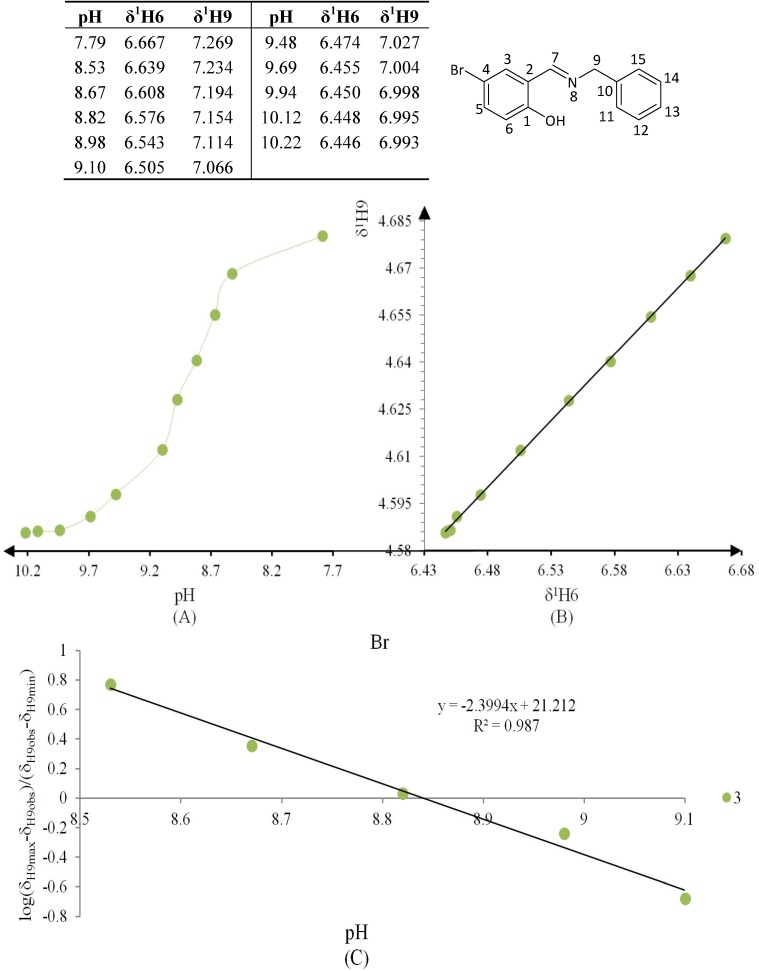
(**A**) titration curve (pH *vs.* δ^1^H9); (**B**) δ-diagram with Perrin model analysis (δ^1^H6 *vs.* δ^1^H9) and (**C**) plot of the semilogarithmic Henderson-Hasselbalch equation (pH *vs.* log[(δ_H9max_ − δ_H9obs_)/(δ_H9obs_ − δ_H9min_)]) of compound **3** (R = Br).

**Figure 9:**
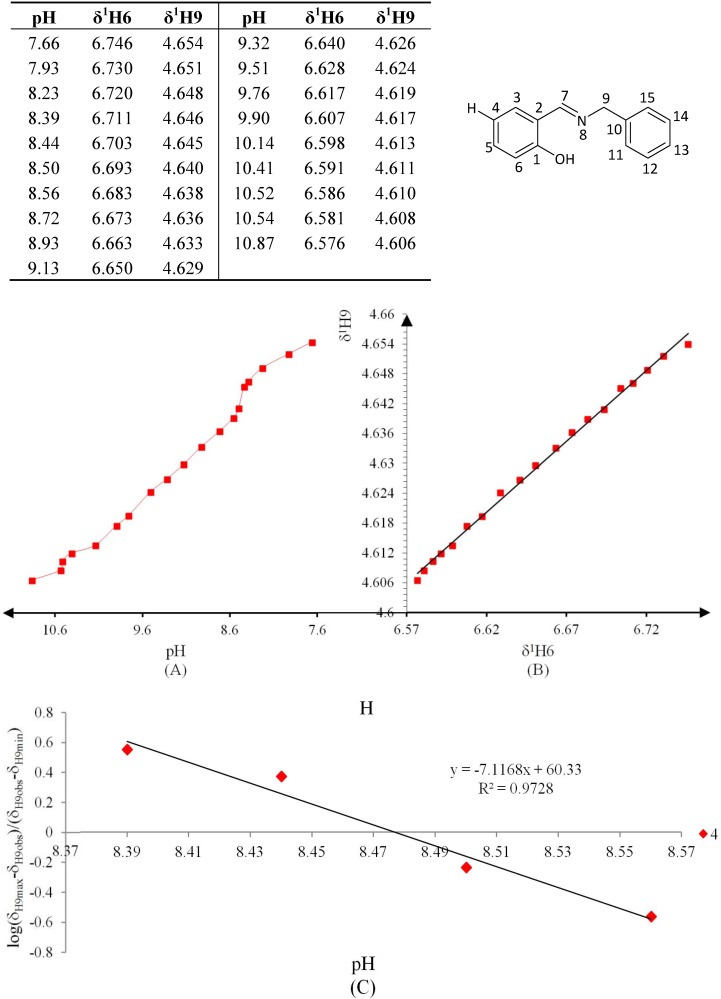
(**A**) titration curve (pH *vs.* δ^1^H9); (**B**) δ-diagram with Perrin model analysis (^1^H6 *vs.* ^1^H9) and (**C**) plot of the semilogarithmic Henderson-Hasselbalch equation (pH *vs.* log[(δ_H9max_ − δ_H9obs_)/(δ_H9obs_ − δ_H9min_)])of compound **4** (R = H).

**Figure 10:**
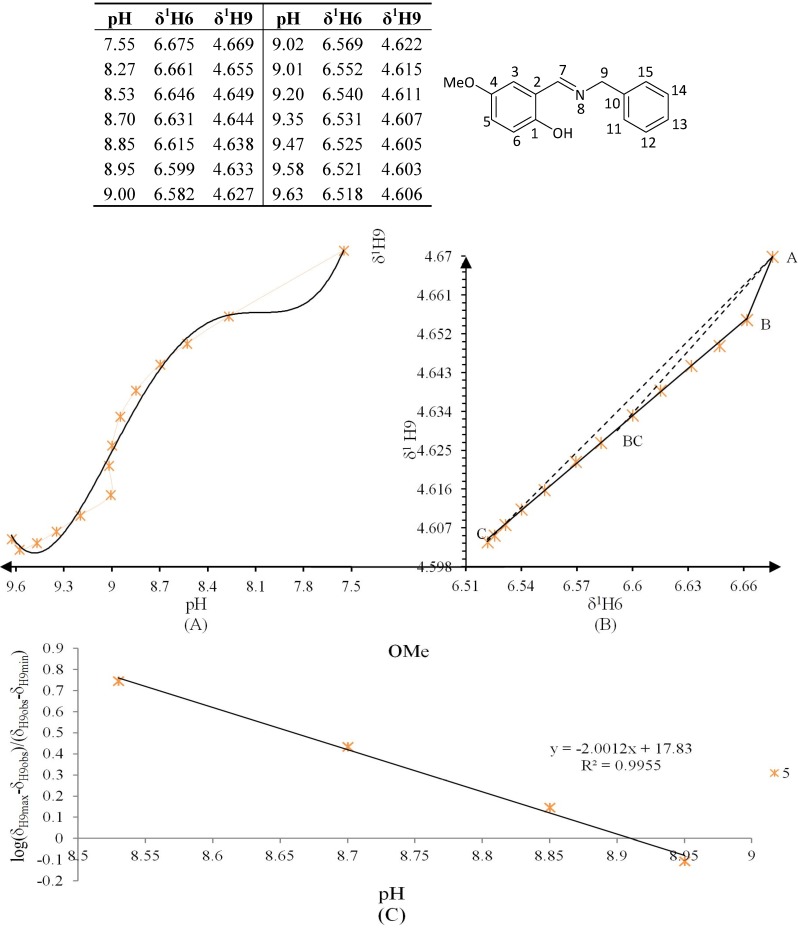
(**A**) titration curve (pH *vs.* δ^1^H9); (**B**) δ-diagram with Polster-Lachmann analysis (δ^1^H6 *vs.* δ^1^H9) and (**C**) plot of the semilogarithmic Henderson-Hasselbalch equation (pH *vs.* log[(δ_H9max_ − δ_H9obs_)/(δ_H9obs_ − δ_H9min_)]) of compound **5** (R = OMe).

**Figure 11:**
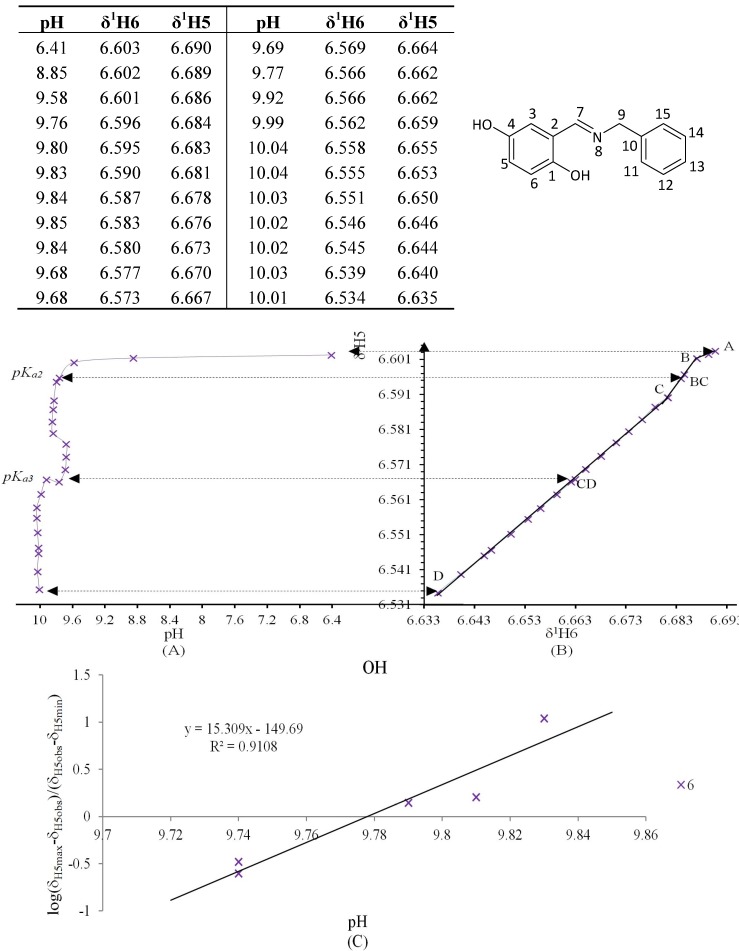
(**A**) titration curve (pH *vs.* δ^1^H5); (**B**) δ-diagram with Polster-Lachmann analysis (δ^1^H6 *vs.* δ^1^H5) and (**C**) plot of the semilogarithmic Henderson-Hasselbalch equation (pH *vs.* log[(δ_H5max_ – δ_H5obs_)/(δ_H5obs_ – δ_H5min_)])of compound **6** (R = OH) calculating the *pK_a2_*. The *pK_a2_* value belong to deprotonation of intramolecular hydrogen bond N∙∙∙H-O, whereas *pK_a3_* correspond to free phenolic C4-OH. For this compound the plot of δ-diagram was done with ^1^H6 *vs.* ^1^H5 chemical shifts, since only this correlation showed a system with three slope changes similar to polyprotic system.

### 3.5. Data Analysis

The calculations were performed on a Microsoft Excel worksheet. The data analysis of the semilogarithmic Henderson-Hasselbalch (Equation (3)) was applied to data that have adapted to this analysis method, using a dependent variable log[(δ_Hmax_ − δ_Hobs_)/(δ_Hobs_ − δ_Hmin_)]; H9 for compounds **1**–**5** and H5 for compound **6**) (HX = H5 or H9) and *pH* as an independent variable for titration curves to find the *pK_a_* values.

Data analysis for the δ-diagram by both Perrin and Polster-Lachmann analysis used H6 and H9 proton chemical shifts for the analysis in δ-diagram, since they are the proton chemical shifts adjacent to NHO intramolecular hydrogen bond and the most affected by deprotonation. Thus in the Perrin Analysis Equation (5) can be written as follows (Equation (7)):

(*δ_H9_ − δ_H9°_*)(*δ_H6^e^_ − δ_H6_*) = ∆*K_NHO_*(*δ_H6_ − δ_H6°_*)(*δ_H9^e^_ − δ_H9_*) (7)

where δ_H9°_ and δ_H6°_ are the chemical shifts from the species at the beginning of the titration, δ_H9_ and δ_H6_ the chemical shifts observed in the course of the titration, δ_H9_^e^ and δ_H6_^e^ are the chemical shifts from species at the end of the titration. Finally in the Polster-Lachmann analysis, the ratio of distances to calculate the Δ*K_NHO_* value is established by the graphic method described by the Gibbs triangle [62,68].

## 4. Conclusions

The study of compounds **1**–**6** by NMR titration in methanol solution, confirmed the predominant tautomeric forms in solution, noting that the NHO prototropic equilibrium is dependent of the substituent and the solvent. The *pK_a_* values obtained using the Henderson-Hasselbalch analysis showed that all compounds are weak acids. The strength and lability of the NHO intramolecular hydrogen bond are consequently affected by the mesomeric and inductive effects exerted by the substituents. The values of the *K_NHO_* equilibrium constant indicate that the equilibrium is slightly shifted to the nitrogen atom when the substituent in the phenyl ring exerts a strong electronic effect, either ED or EW (R = NO_2_, Cl, OMe and OH), and to the oxygen atom when Br or H in CD_3_OD solutions. Nevertheless the *ΔK_NHO_* values close to the unit, highlight that the proton is in the middle of both basic sites (O^−^∙∙∙H∙∙∙N^+^), in contrast to what is found in DMSO-*d*_6_ solutions, where NMR data is in agreement with the neutral N∙∙∙H–O tautomer for most of the compounds except for the nitro derivative which is in the zwitterion ^+^N–H∙∙∙O form. Finally, we have demonstrated the simplicity, accuracy and versatility of both the Perrin and Polster-Lachmann analysis applied to the study of intramolecular hydrogen bonds.
